# *PA*lliative *C*are in chronic *K*idney di*S*ease: the PACKS study—quality of life, decision making, costs and impact on carers in people managed without dialysis

**DOI:** 10.1186/s12882-015-0084-7

**Published:** 2015-07-11

**Authors:** Helen Rose Noble, Ashley Agus, Kevin Brazil, Aine Burns, Nicola A Goodfellow, Mary Guiney, Fiona McCourt, Cliona McDowell, Charles Normand, Paul Roderick, Colin Thompson, A. P. Maxwell, M. M. Yaqoob

**Affiliations:** School of Nursing and Midwifery, Queen’s University Belfast, Medical Biology Centre: 97 Lisburn Rd, BT9 7BL Belfast, UK; Northern Ireland Clinical Trials Unit, 1st Floor Elliott Dynes Building, Royal Hospitals, Grosvenor Road, Belfast, BT12 6BA UK; Royal Free Hospital, Pond Street, London, NW3 2QN UK; Trinity College Dublin, 3–4 Foster Place, Dublin 2, Ireland; University of Southampton, Mailpoint 805, C floor, South Academic Block, Southampton General Hospital, Southampton, SO166YD UK; School of Medicine, Dentistry and Biomedical Sciences, Queens University Belfast & Regional Nephrology Unit, Belfast City Hospital, Belfast HSC Trust, Belfast, UK; William Harvey Research Institute, Queen Mary University of London, London & Renal Unit, The Royal London Hospital, London, E1 1BB UK; Kidney Patient Association, Northern, Ireland UK

**Keywords:** Carers, Conservative kidney management, End-of-life, Mixed methods research, Palliative care, Quality of life renal

## Abstract

**Background:**

The number of patients with advanced chronic kidney disease opting for conservative management rather than dialysis is unknown but likely to be growing as increasingly frail patients with advanced renal disease present to renal services. Conservative kidney management includes ongoing medical input and support from a multidisciplinary team. There is limited evidence concerning patient and carer experience of this choice. This study will explore quality of life, symptoms, cognition, frailty, performance decision making, costs and impact on carers in people with advanced chronic kidney disease managed without dialysis and is funded by the National Institute of Health Research in the UK.

**Methods:**

In this prospective, multicentre, longitudinal study, patients will be recruited in the UK, by renal research nurses, once they have made the decision not to embark on dialysis. Carers will be asked to ‘opt-in’ with consent from patients. The approach includes longitudinal quantitative surveys of quality of life, symptoms, decision making and costs for patients and quality of life and costs for carers, with questionnaires administered quarterly over 12 months. Additionally, the decision making process will be explored via qualitative interviews with renal physicians/clinical nurse specialists.

**Discussion:**

The study is designed to capture patient and carer profiles when conservative kidney management is implemented, and understand trajectories of care-receiving and care-giving with the aim of optimising palliative care for this population. It will explore the interactions that lead to clinical care decisions and the impact of these decisions on informal carers with the intention of improving clinical outcomes for patients and the experiences of care givers.

## Background

Improving quality of death and access to palliative care is of international concern and in some countries strategies to provide a dignified death in those who are dying have been developed [1–4]. In addition there is a need to ensure those with non-malignant disease have equitable access to appropriate and supportive care services towards the end-of-life [5]. The number of people living with end-stage kidney disease (ESKD) has increased in part due to improved access of an aging population to ESKD care but also due to a higher prevalence of risk factors for chronic kidney disease such as diabetes and hypertension [6, 7]. Worldwide, over 1.4 million people receive renal replacement therapy [8] and the incidence is growing annually by approximately 8 % [9]. In the UK there were 54,824 adult patients receiving renal replacement therapy in 2013 [10]. The mean annual cost of dialysis per patient is estimated at £27 000 [11]. Provision of treatment for ESKD is predicted to consume approximately 2 % of the annual National Health Service budget [12] influenced by disproportionate numbers of older, frailer, dependent patients. Older people with advanced chronic kidney disease have increasing prevalence of co-morbidities [13] and high mortality with a median 2.5 life years remaining for those over 75 years [14]. In addition treatment withdrawal accounts for ∼ 20 % of overall deaths [15]. Evidence is emerging that dialysis may be of little value, in terms of survival benefit and quality of life, to some frailer patients with multiple co-morbid conditions and poor functional status [16, 17]. This has led to questioning of the suitability of renal replacement therapy for ESKD in this population [18] and the impact on quality of life [19].

ESKD includes those patients who have reached stage 5 chronic kidney disease (estimated glomerular filtration rate ≤15 mL/min/1.73 m^2^ as measured using the Modification of Diet in Renal Disease equation (MDRD) formula) [20]. ESKD is a life-limiting condition associated with substantially increased risks of morbidity and mortality. The glomerular filtration may continue to fall in patients with ESKD until a point is reached where dialysis would normally be initiated to maintain life. In a number of renal units in the UK, patients with ESKD are offered an alternative treatment to dialysis or transplantation known as conservative kidney management where a palliative care approach is adopted and supportive care provided by the multidisciplinary team often in liaison with the community team and general practitioner (GP). Deciding when to withhold dialysis in this population and provide conservative kidney management as an alternative requires thorough ethical deliberation and complex decision-making. Some patients may not benefit form dialysis but there is limited evidence to guide patients, carers and staff when making this important decision. There are few service models designed to support this group and little known about how they can be best managed. Ideally, clinicians should be able to accurately distinguish between a patient who will do well on dialysis and a patient who will do poorly; however, any attempt to define such a population has been largely unsuccessful [21]. Some studies have explored age [17], functional status [22], and comorbidity burden [23] as predictors of survival but the development of a criterion score to select people for dialysis has not been developed and individualized assessment is always necessary [24]. Informing that assessment with good quality research centred on patient and carer experiences is still required. When these complex decisions are made, the way in which conclusions are made are difficult to extract, teach and embed in practice. A recent thematic synthesis of qualitative studies [25] described patients with ESKD and their caregiver perspectives on conservative management and end-of-life care and found only five studies [13, 26–29] offering a limited insight into quality of life (QOL) and decision making in those managed without dialysis with no exploration of changes over time. A systematic review of conservative management [30] identified literature in this field as widely distributed and difficult to uncover with database search strategies. Findings were limited and preliminary in nature comparing small groups of patients. Results were not stratified by age or comorbidities and the authors suggest that in patients who opt for non-dialytic management of their kidney disease, guidelines are still required to determine the best clinical practice in this area. There appears to be no studies that explore economic aspects of conservative management in ESKD.

Prospective, randomised trials of dialysis versus conservative kidney management for ESKD are neither ethically justifiable nor practical and therefore only observational studies are available to inform practice [31]. A recent study by Da Silva-Gane and colleagues [32] attempted to compare QOL in patients with advanced chronic kidney disease in those opting for dialysis versus conservative kidney management. This small study, did not investigate clinical decision making, resource use or impact of conservative kidney management on carers. The authors concluded that those opting for conservative kidney management may maintain a better QOL compared to dialysis but higher levels of anxiety were seen in the conservative kidney management patients. The study was limited by major demographic and clinical differences between individuals and the modality groups.

To facilitate improved decision making for individuals with ESKD, accurate information is required for a number of issues including the expected QOL for patients with ESKD, the potential impact a decision not to dialyse may have on the QOL of carers; resource use and costs of conservative care for ESKD and the factors that influence decision making from a patient/carer and health care practitioner perspective [33–36]. Unfortunately there is a dearth of research in this area. The present multicentre study is designed to capture patient and carer profiles when conservative kidney management is implemented and to understand the trajectories of care-receiving and care-giving. It will explore the interactions that lead to clinical care decisions and the impact of these decisions on carers with the intention of improving clinical outcomes for patients and the care giver experience. The economic analysis of conservative kidney management will facilitate greater transparency in relation to resource allocation processes for persons with chronic kidney disease. The purpose of this article is to describe the design of the present study and inform others on the possibilities of performing end-of-life care research in advanced chronic kidney disease. The prospective and longitudinal nature of the design will be discussed. In addition the explanation of the methodology will serve as a comprehensive orientation for the methods section of publications arising from the research.

The aim of the study is to measure and describe longitudinally QOL, symptoms, cognition, frailty and performance in persons with ESKD being managed without dialysis and the impact of these issues on their carers. In addition, the study will analyse decision making related to conservative care for ESKD together with the costs of conservative kidney management.

### Study objectives

#### Patient focused

- To measure and describe longitudinally patient reported outcomes including QOL, satisfaction in decision-making and costs in patients receiving conservative kidney management (palliative care).
- To measure and describe longitudinally changes in cognition, frailty and performance in patients receiving conservative kidney management
- To measure and describe the associated health and social care costs of patients receiving conservative kidney management.

#### Carer focused

- To measure and describe longitudinally QOL for carers of patients receiving conservative kidney management.
- To measure and describe the subjective and objective burden of providing informal care in carers, loss of earnings and the opportunity costs of providing such care

#### Renal physicians/clinical nurse specialist focused

- To explore the decision making process that precedes referral for conservative kidney management in relation to patient satisfaction in decision making.

## Methods/Design

The research study is a Post-Doctoral Fellowship funded by the National Institute of Health Research in the UK. It is a mixed method study and includes quantitative and qualitative components [37]. In the quantitative component a longitudinal survey of QOL, symptoms, cognition, frailty, performance, satisfaction with decision-making, health service use of patients and associated costs, subjective burden and QOL of carers, loss of earnings and the opportunity cost of providing informal care will be explored. In the qualitative component the decision making process with patients and carers that precedes referral to conservative kidney management will be explored with renal physicians and/or clinical nurse specialists in relation to patient satisfaction in decision making. The longitudinal nature of the study will be explained to participants. Some people may change their mind regarding their selected treatment option and commence dialysis although this is unusual in clinical practice. If this happens the patient and carer data will continue to be collected in order to examine how QOL might increase or decrease with a switch of modality.

The study is multicentre across ten sites in Northern Ireland, England and Scotland. Each site offers conservative management as a treatment option to patients who have reached ESKD. All patients who make the decision not to embark on dialysis will be approached to take part in the study. Their main carer will be asked to ‘opt-in’ to the study with the patient’s consent.

### Study approvals

The study protocol has been approved by the Office for Research Ethics Committees Northern Ireland (ORECNI) (REC reference 14/NI/0057; 14 May 2014) and research governance approval has been granted by the five Health and Social Care Trusts in Northern Ireland and three acute hospital trusts in England, UK with two others in progress in London, England and Glasgow, Scotland. The study is registered with the International Standard Randomised Controlled Trial (ISRCTN) (ISRCTN06857980) [38]. It is sponsored by Queens University Belfast and the Western Health and Social Care Trust, and has been adopted by the Northern Ireland Clinical Research Network (Renal). It is supported by the Northern Ireland Clinical Trials Unit, a UK Clinical Research Collaboration registered clinical trials unit.

### Study population

#### Patients

To be eligible for recruitment into the study patients must be aged 18 years or older; have reached stage 5 chronic kidney disease with estimated glomerular filtration rate ≤15 mL/min/1.73m^2^as measured by the MDRD formula [20]; and have made a confirmed decision for conservative kidney management, i.e. management without dialysis or other renal replacement therapy. Patients lacking capacity to give consent to participate will be excluded. Capacity for consent to participate will be assessed in collaboration with the supervising nephrologists. Non-English speaking patients or those who do not adequately understand verbal or written information will be excluded.

#### Carers

To be eligible for recruitment into the study participants must identify as the primary carer for the patient who has made a confirmed decision for conservative kidney management. They must be over 18 years and the patient must agree that the carer can be approached to participate. Carers can only participate in the study if they ‘opt in’ to study by making contact with the Research Nurse. Carers who lack capacity to give consent to participate in the study will be excluded. Non-English speaking carers will also be excluded.

#### Renal physicians/clinical nurse specialists

Renal physicians and clinical nurse specialists (CNS) recruited to the study must have experience of managing clinical consultations with patients who have stage 5 chronic kidney disease and are opting for conservative kidney management. Those clinicians and nurses without experience of managing consultations with patients who opt for conservative kidney management and who are not nephrologists or renal nurses will be excluded.

### Recruitment

#### Patients

**Fig. 1:**
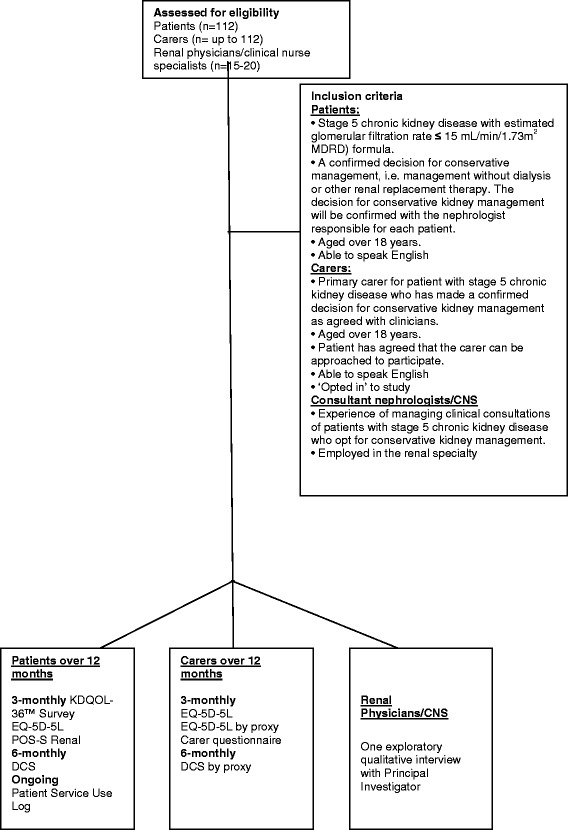
CONSORT diagram showing the flow of participants through each stage of study

Recruitment will take place over 18 months and data collection will continue for another 12 months or until death. The initial treatment decision is made in clinic with a renal physician or a CNS. Once the decision is made Research Nurses at each site will liaise with renal physicians or CNS and use the inclusion/exclusion criteria to identify potential participants. All patients across the ten study sites who have made a decision not to initiate dialysis and meet the study inclusion criteria will be sent written information about the study (see Fig. 1). They will be contacted to see if they are willing to take part in the study. If willing to participate written informed consent will be obtained at their next clinic visit (Baseline). This should take place within 3 months after the initial decision not to embark on dialysis.

If patients wish they can complete questionnaires themselves, with the nurses or with their carers. The way in which the data are captured (self-completion or help from staff member or carer) will be noted on the front sheet of the questionnaires.

#### Carers

With the patient’s agreement, and if the patient is happy for the carer to participate in the study, information will be sent to the patient to give to their carer. Carers will be asked to contact the Research Nurse if they are interested in ‘opting-in’ to the study. If a carer is interested in participating they will be invited to attend the patient’s next clinic appointment so that informed consent can be obtained and baseline data collected (see Fig. 1). If the carer is unable to attend this clinic visit then the Research Nurse will send a consent form and baseline data collection tools to their home. The carers will be asked to return the consent form to the hospital. Information on how to complete the tools will be given over the telephone. Carers can either complete the tools themselves or with the assistance of a Research Nurse.

#### Renal physicians/clinical nurse specialists

Renal physicians and CNS from each of the ten study sites who fulfil the inclusion criteria will be identified through the lead nephrologists or senior renal managers for each study site. The renal physicians and CNS will be invited to take part in a semi-structured qualitative interview, conducted to explore their experience of counselling a patient who ultimately declines dialysis (see Fig. 1). They will be approached by the Chief Investigator of the study via email. Written informed consent will be obtained prior to the interview taking place. Recruitment will continue until data are saturated [39].

#### Sample size

The primary outcome is to measure the true mean QOL at 3 months using the EQ-5D-5L Visual Analogue Scale (EQ-VAS). The EQ-VAS asks patients to place their health on an interval scale from 0–100 where 0 is the worst state they can imagine and 100 is the best. Using a standard deviation estimate of 18 from a similar study (Nephrol Dial Transplant (2004) 19: 1594–1599),with 100 individuals in our sample we would calculate a 95 % confidence interval for the true mean which would be of width 7.2 units, i.e. 95 % of the time we would estimate the true mean within plus or minus 3.6 units. With an estimated death/dropout rate at 3 months of 10 %, a total of 112 patients are required. For the exploratory qualitative interviews with nephrologists and CNS, in order to achieve data saturation when no new patterns emerge, a sample of approximately 15–20 will be recruited until data saturation reached [39].

#### User involvement

The proposal has been developed in collaboration with members of the Kidney Research and Education Initiative (KREI), established in 2010 to broaden collaborative working between renal research groups and renal education teams to ensure involvement with patients, carers, academics, clinicians and researchers [40]. In addition, we collaborated with the Northern Ireland Kidney Patient Association (a self-help group run by patients with ESKD and their carers) to help set the study aims and objectives. All those involved in this research proposal have personal knowledge and experience of kidney disease and offer differing perspectives to the research team. Regular feedback meetings will be arranged on the progress of the research for comment and scrutiny with a particular focus on ethics, documentation (for example in questionnaires, and information leaflets) and methodology. This shared decision-making will strengthen the partnership between patients, clinicians and researchers involved in the research. Several of the questionnaires were reviewed by members of the Northern Ireland Kidney Patient Association and they provided constructive feedback on feasibility of the study to the research team. The data collection tools were then piloted with 5 patients and 5 carers aged over 70 years attending a renal clinic to test for their clarity, ease of use and time burden. Minor changes were made to the tools to be used to collect economic information from patients and carers. All patients, carers, renal physicians and CNS will have the right to withdraw from the study at any time.

### Data collection

#### Patients

At baseline the research nurse will collect patient demographics through a review of clinical records at study entry (Table 1). Data collected will include age, gender, ethnicity (using UK Office for National Statistics categories), marital status, disease severity (as measured by eGFR) and primary renal disease; comorbidity using the Davies Comorbidity Score. Information will also be obtained on smoking habits of the patient, their diabetes status and on the presence or absence of vascular disease (ischaemic heart disease, angina, myocardial infarction, percutaneous coronary intervention, coronary artery bypass graft, peripheral vascular disease and cerebrovascular accidents). Biochemical data at time of entry to the study will be recorded including serum creatinine, albumin, calcium, phosphate, parathyroid hormone, alkaline phosphatase, liver function tests, haemoglobin level and urine protein creatinine ratio. The research nurse will also be asked to document a ‘Yes’ or ‘No’ answer to the question, ‘would you be surprised if the patient dies within 6 months?’ They will also complete the 9-point Clinical Frailty Scale, 6 item Cognitive Impairment Test (6CIT) and Palliative Performance Score (PPS). Detail from patients’ hospital notes will be collected on who made the decision to accept conservative management. The Decisional Conflict Scale (DCS) will be completed at baseline and 6 monthly (see Table 2).

**Table 1:** Schedule of Assessments

Table 1	Screening	Visit (1)	Visit (2)	Visit (3)	Visit (4)
	Baseline	3 months	6 months	9 months	12 months
PATIENT					
Informed Consent	X				
Inclusion/Exclusion Criteria	X				
Demographic Data	X				
Detail from patient’s hospital notes on who made decision to accept CM	x				
Davies Comorbidity Score	x	x	x	x	x
9-point Clinical Frailty Scale	x	x	x	x	x
6 Item Cognitive Impairment Test (6CIT)	x	x	x	x	x
Functional performance using the PPS	x	x	x	x	x
KDQOL	x	x	x	x	x
EQ-5D-5L	x	x	x	x	x
POS-S Renal	x	x	x	x	x
DCS	x		x		x
Patient Service Use Log	x	x	x	x	x
Serum creatinine, albumin, calcium, phosphate, parathyroid hormone, alkaline phosphatase and haemoglobin level	x	x	x	x	x
Urine protein creatinine ratio	x	x	x	x	x
Liver function tests	x	x	x	x	x
Smoking habits of patient	x	x	x	x	x
Smoking habits of other people living in household	x	x	x	x	x
Diabetes and diabetic comorbidity check	x	x	x	x	x
Check for Ischemic heart disease, angina, myocardial infarction, percutaneous coronary Intervention, coronary artery bypass graft, peripheral vascular disease, cerebrovascular accidents in all patients.	x	x	x	x	x
Answer question ‘Would you be surprised if patient dies within 6 months?	x	x	x	x	x
CARER					
Informed Consent	x				
Inclusion/Exclusion Criteria	x				
EQ-5D-5L	x	x	x	x	x
EQ-5D-5L for the patient by the carer (i.e. by proxy)	x	x	x	x	x
DCS	x		x		x
Carer baseline questionnaire	x				
Follow up carer questionnaires		x	x	x	x
RENAL PHYSICIAN/ CLINICAL NURSE SPECIALISTS	At one point during study				
Qualitative interview

**Table 2:** Data collection tools for PACKS study

**Tools for use with patient**
*Kidney Disease QOL-36™* Quality of life of patients will be measured using the Kidney Disease QOL-36™ Survey (KDQOL) [48], a well validated tool in kidney disease which has demonstrated good test-retest reliability on most dimensions (includes general health, activity limits, ability to accomplish desired tasks, energy level, and social activities). Symptoms and problems will also be assessed (questions 17–28) and include items about how bothered a respondent feels by sore muscles, chest pain, cramps, itchy or dry skin, shortness of breath, faintness/dizziness, lack of appetite, feeling washed out or drained, numbness in the hands or feet and nausea. Anxiety and depression will also be assessed using the KDQOL-36™ and is measured within the mental component of the tool (questions 1–12).
*EQ-5D-5L* The EQ-5D [49] is the National Institute of Health and Clinical Excellence’s (NICE) preferred method of measuring health effects in economic evaluations and it has shown to be a valid instrument for the measurement of health status in renal patients. The use of the new 5 level version, EQ-5D-5L, is also advocated by NICE. It consists of a descriptive system and a visual analogue scale. The EQ-5D-5L will be self-completed by the patients or the Research Nurse and also completed for the patient by the carer (i.e. by proxy). The inter-rater agreement can then be assessed.
*POS-S Renal* The POS-S Renal was developed in 2011 and is used as a tool to monitor progress in individual symptoms. It is a brief tool, primarily aimed at patients with advanced disease [50].
*6 Item Cognitive Impairment Test (6CIT)* The 6 Item Cognitive Impairment Test (6CIT) Kingshill Version 2000® was developed in 1983 [51] and is a useful dementia screening tool in Primary Care. The tool will be used to identify cognitive impairment and changes over time during the course of the study.
*9-point Clinical Frailty Scale* Frailty will be studied using the 9-point Clinical Frailty Scale [52]. The Clinical Frailty Scale© has performed better than measures of cognition, function or comorbidity in assessing risk for death.
*Palliative Performance Scale (PPS)* The Palliative Performance Scale [53] uses five observer-rated domains correlated to the Karnofsky Performance Scale (100–0). The PPS is a reliable and valid tool and correlates well with actual survival and median survival. It has been found useful for purposes of identifying and tracking potential care needs of palliative care patients, particularly as these needs change with disease progression.
*Decisional Conflict Scale (DCS)* This will be used to explore satisfaction with decision making from a patient perspective. The scale measures uncertainty and difficulties in the decision making process [54]. The 16 item version measures four domains: a) uncertainty in choosing options; b) unsupported in decision making; c) feeling informed; d) decision is consistent with values. The instrument demonstrates satisfactory reliability and good construct validity [55]. It has been used extensively in the United Kingdom. It will be used with patients at baseline, 6 and 12 months.
*Patient Log* Questionnaires have been developed using items from the Annotated Cost Questionnaire [56] and the iMTA Valuation of Informal Care Questionnaire [57]. These will measure healthcare resource utilisation (and associated costs) by patients.
**Tools for use with carer**
*EQ-5D-5L* Carer quality of life will be measured using the EQ-5D-5L detailed above. Carers will also use the EQ-5D-5L to assess the patient’s quality of life.
*Decisional Conflict Scale (DCS)* Carers views on the patient’s Satisfaction with Decision Making will also be explored using the DCS. It will be used with patients and carers at baseline and at 6 months.
*Carer questionnaire* Care-related costs to carers using questionnaires will be captured whilst patient included in study or study end. Cost measures will include caregiver’s lost income and out-of-pocket expenditures for formal care-giving services.
**Renal Clinicians/CNS**
*Qualitative interviews* Individual semi-structured interviews with renal physicians/CNS will focus on the decision making process with patients and carers that precedes referral to conservative kidney management. Experiences of physicians related to counselling a patient who makes the decision not to commence dialysis will be captured (For interview guide based on the Decisional Conflict Scale (DCS) (see Table 3)

#### Carers

At baseline and 3-monthly for 12 months, carers will be asked to complete, the EQ-5D-5L whilst the patient is included in study or 3 months after death of patient or study end; the EQ-5D-5L for the patient by proxy and a questionnaire to measure the subjective and objective burden of providing care, loss of earnings and the opportunity costs of providing care. The DCS will be completed at baseline and 6 monthly (see Table 2).

### Renal physicians/clinical nurse specialists

Exploratory semi-structured qualitative interviews with consultant nephrologists and CNS will be undertaken by the Chief Investigator over the period of the study to explore the decision making process that precedes referral to conservative kidney management in relation to patient satisfaction in decision making. Questions can be viewed in Table 3.

**Table 3:** Interview guide for qualitative interviews with renal physicians/clinical nurse specialists

Do you feel your patients know what treatment options are available to them?
Do you think they know the benefits of each option?
Do you think they know the risks and side effects of each option?
Do you think they are clear about which benefits matter most to them?
Do you think they are clear about which risks and side effects matter most to them?
Do you think they are clear about which is more important to them (the benefits or the risks and side effects).
Do you think they have enough support from others to make a choice?
Do you think they choose without pressure from others?
Do you think they have enough advice to make a choice?
Do you think they are clear about the best choice for them?
Do you think they feel sure about what to choose?
Do you think the decision is easy for them to make.
Do you think they feel they make an informed choice?
Do you think their decision shows what is important to them?
Do you expect them to stick with their decision?
Do you think they are satisfied with their decision?
What guidelines/decision tools do you use with your patients when helping them make treatment decisions?

#### Outcome measures

The primary outcome of the study is QOL of patients at 3 months measured using the EQ-VAS.

The range of data collection tools can be viewed in Table 2

### Secondary outcome measures

#### Patients

- Changes in QOL, symptoms, anxiety and depression in patients using the Kidney Disease QOL (KDQOL) tool, EQ-5D-5L (which includes the EQ-VAS) and Palliative Outcome Scale - Symptoms (POS-S) Renal 3-monthly over 12 months
- Changes in cognition and frailty status in patients using the 6 Item Cognitive Impairment Test (6CIT) and the 9-point Clinical Frailty Scale 3-monthly over 12 months
- Changes in Performance in patients using the Palliative Performance Scale (PPS) 3-monthly over 12 months
- Patient Satisfaction in Decision Making using the Decisional Conflict Scale (DCS) at baseline
- Measurement of health and social care costs of patients receiving conservative kidney management using a Patient Service Use Log 3-monthly over 12 months
- Calculation of the number (%) of patient deaths 3-monthly and time to death.

#### Carers

- Carer observation of patient’s Satisfaction in Decision Making using the Decisional Conflict Scale (DCS) at baseline
- Changes in QOL of carers using the EQ-5D-5L and Carer QOL 3-monthly over 12 months
- Carers assessment of patient QOL using the EQ-5D-5L by proxy 3-monthly over 12 months
- Subjective and objective burden of providing informal care in carers, loss of earnings and the opportunity costs of providing informal care using items from the iMTA Valuation of Informal Care Questionnaire 3 monthly over the 12 months.

### Renal physicians/clinical nurse specialists

- Exploration of the decision making process that precedes referral to conservative kidney management in relation to patient satisfaction with renal physicians/CNS via exploratory qualitative interviews.

The range of data collection tools can be viewed in Table 2

#### Quantitative data analysis

Descriptive statistics will be used to summarise baseline demographics and the questionnaire data from patients and carers.

#### Primary outcomes

The mean and 95 % CI for the EQ-VAS will be calculated. Additional exploratory analyses will be used to compare the mean EQ-VAS at 3 months between categories of categorical variables (such as gender, marital status comorbidity) using ANCOVA adjusting for baseline scores. The association between EQ-VAS nd other continuous variables will be investigated using simple linear regression with EQ-VAS at 3 months as the dependent variable. Multiple linear regressions will also be used to investigate associations with comorbidities, age, gender, marital status, severity of symptoms and depression to allow adjustments for confounding. As the study is not powered to detect statistically significant differences in EQ-5D-5L VAS all results will be interpreted with caution. Statistical significance will be assumed for P values 0.05.

#### Secondary outcomes

Descriptive summaries of patient and carer questionnaire data at 3, 6, 9 and 12 months will be tabulated and presented graphically where appropriate. The number (%) of deaths at 3, 6, 9 and 12 months will be tabulated. Time to death data will be investigated using Kaplan Meier curves. The log rank test statistic will be used to compare categorical variables and Cox proportional hazards model for continuous variables where appropriate.

#### Qualitative data analysis

In the exploratory qualitative aspect of the study, coding of qualitative data (renal physician/CNS interviews) will be assisted with the use of NVivo version 10, qualitative software to organise, store and retrieve data [41]. Renal physician/CNS interviews will be recorded by the researcher using a digital recorder and subsequently professionally transcribed by a transcription company previously employed to do similar work. Electronic versions of diary transcripts will be saved and imported into the software programme to enable computer-assisted coding analysis. This iterative process will be guided by an approach described by de Wet and Erasmus [42]. Their approach draws on grounded theory techniques including first level coding and pattern coding and the development of relationships in the data.

#### Economic evaluation

A partial economic evaluation in the form of a cost-outcome description of conservative kidney management will be performed. Each patient’s health and social care resource use and quality of life measurements over the 12 month study period will be collected as stated under Data Collection. Unit costs will be applied to the quantity of resource use for each patient and these will be obtained from national sources where possible. Utilities for the calculation of quality adjusted life years (QALYs) will be obtained using responses on the EQ-5D-5L.

Care-related costs for the carers will also be explored. Multiple regression methods will be used to examine patient and carer factors which are potentially associated with their costs and to adjust for potential confounders. Although the cost effectiveness of conservative kidney management compared with dialysis cannot be established within the current single arm study design, the estimation of the costs and outcomes of conservative kidney management will allow comparisons to be made with similar estimates for dialysis already in the literature.

### Study quality, monitoring and ethical considerations

To ensure the research quality and data is of a high standard rigorous study conduct and monitoring procedures will be undertaken to ensure any problems are identified and managed. Regular Trial Management Group meetings with the Northern Ireland Clinical Trials Unit have taken place and will continue and an Advisory Group of experts has met on two occasions and will continue to meet throughout the course of the study. Formal monitoring of study procedures will take place at least once per year over the course of the study at each site and as required.

Some patients in the study will be close to death. All attempts will be made to ensure the best interests of patients and carers are taken into account. Permission to gain access to patients will first be sought from staff. There is the potential for patients and carers to feel coerced into the study due to the status of the research nurses who are assisting with recruitment and data collection. This could intimidate participants into agreeing to participate in the study [43]. One way of attempting to shift some of the power differentials is to explain honestly to patients and carers that their care will not be compromised if they do not agree to be involved in the study and it is entirely their decision. It will be necessary to look for signs of fatigue or discomfort and to curtail any assessments as necessary. Consideration will also need to be given to issues such as the length of assessment and time of day when it takes place. Information (written and oral) will need to take into account cognitive abilities and participants’ preferred way of assimilating information and may need to be adapted appropriately e.g. some patients may need to be spoken to slowly or need certain words explained. One view of dying people is that they are in need of protection and should not be approached to be involved in research [44]. An alternative view is that everyone, whether dying or not should make the choice for themselves. Throughout the study patients will be fully informed regarding the research process in order to make appropriate decisions and retain the right to withdraw at any time without care being compromised.

## Discussion

This study has been designed to explore QOL, symptoms, cognition, frailty, performance, decision making, costs and impact on carers in people managed without dialysis. Little is known about QOL in those with ESKD who accept conservative kidney management. In addition, there is limited information on this group related to changes in symptoms, frailty and cognition over time. The study will also explore satisfaction with decision making for patients and the costs of this conservative kidney care programme. The impact on carers is virtually unknown. The present study involves an under researched group particularly in relation to their QOL experiences, symptom burden and changes in symptoms over time. Although usually a frail group with limited life expectancy [45] cognition, frailty and performance are also poorly explored in this population.

Davison [46] has described the methodological challenges for end-of-life research in patients with chronic kidney disease and highlighted the difficulties of retrospective research which draws on limited, routinely collected data. Prospective designs allow for additional collection of patient-derived measures using questionnaires. A prospective design, as in the present study, allows for observation of the end-of-life experience and possible dying process. To date, many studies exploring the end-of-life experience have relied on cross-sectional or retrospective designs although the importance of prospective, longitudinal research in those with chronic disease has been promoted [47]. The identified sample size and the involvement of ten study sites will help improve reliability and generalisability of the findings. In addition the costs associated with conservative management end-of-life care in ESKD, previously unexplored [46], will be investigated. To support translation of findings from the study to clinical practice, dissemination to relevant health professionals and user groups will take place via national and international conference presentations and publication in high impact peer-reviewed journals.

The key contribution of this study will be an assessment of the needs of patients (and carers) managed without dialysis. Findings from this study can lead to development of new healthcare policies and embedded within clinical practice once supportive interventions have been tested and evaluated. Patients will benefit as more accurate information on conservative kidney management, including the predicted QOL without dialysis, can be provided in the counselling process as patients make treatment decisions. Impact on carers will be acknowledged as a first step in developing a health-improvement programme for carers of this patient group. Associated costs of conservative kidney management will be identified allowing for comparison with other ESKD treatment modalities. In the proposed project, knowledge transfer to a broader audience will be facilitated by various mechanisms including carer and user organisations and clinically based staff. The protocol has been developed with clinicians, users and carers with a key benefit of strengthening links between researchers, practitioners, policy makers and voluntary sectors to ensure new knowledge is translated into practice. These links will be developed and utilised to disseminate the results of the proposed programme of research, to raise awareness of developments and research priorities in renal care and end-of-life practice.

## Abbreviations

ESKD: end-stage kidney disease
MDRD: Modification of Diet in Renal Disease equation
QOL: Quality of life
ORECNI: Office for Research Ethics Committees Northern Ireland
CNS: Clinical nurse specialist
VAS: Visual Analogue Scale
KREI: Kidney Research and Education Initiative
6CIT: 6 item Cognitive Impairment Test
PPS: Palliative Performance Score
DCS: Decisional Conflict Scale
KDQOL: Kidney Disease QOL
QALYs: Quality adjusted life years
